# Exploring Intraosseous Meningiomas: Case Series and Literature Insights from a Tertiary Care Hospital in Pakistan

**DOI:** 10.12669/pjms.41.13(PINS-NNOS).13488

**Published:** 2025-12

**Authors:** Nasruddin Ansari, Samra Majeed, Syed Shahrukh Hassan Rizvi, Adeel-Ur Rehman, Talha Sajid, Abdul Majid

**Affiliations:** 1 Nasruddin Ansari, MBBS.Resident Neurosurgery, Neurosurgery Punjab Institute of Neurosciences (PINS), Lahore, Pakistan; 2 Samra Majeed, MBBS, FCPS, Associate Professor of Neurosurgery, Neurosurgery Punjab Institute of Neurosciences (PINS), Lahore, Pakistan; 3 Syed Shahrukh Hassan Rizvi, MBBS, FCPS. Senior Registrar Neurosurgery, Neurosurgery Punjab Institute of Neurosciences (PINS), Lahore, Pakistan; 4 Adeel Ur Rehman, MBBS. Resident Neurosurgery, Neurosurgery Punjab Institute of Neurosciences (PINS), Lahore, Pakistan; 5 Talha Sajid, MBBS. Resident Neurosurgery, Neurosurgery Punjab Institute of Neurosciences (PINS), Lahore, Pakistan; 6 Abdul Majid, MBBS, FCPS. Professor and Head of the Department of Neurosurgery Unit-III, Neurosurgery Punjab Institute of Neurosciences (PINS), Lahore, Pakistan

**Keywords:** Case series, Craniofacial Bones, Meningioma, Meningioma/diagnostic imaging, Primary Intraosseous Pakistan, Skull Neoplasms/surgery

## Abstract

This study is a retrospective case series exploring the clinical and radiological parameters with surgical approach and its outcome for Intraosseous Meningioma (IM), a rare benign tumor primarily affecting the craniofacial bones. It was conducted at the Department of Neurosurgery Unit-III, Punjab Institute of Neurosciences (PINS) from January 2022 to December 2024. The study included five surgically treated patients through non-probability consecutive sampling. A descriptive analysis was done for clinical-radiological and outcome-based variables. The cohort included four females and one male, with a mean age of 34±15.77 years. Most presented with painless progressive swelling, predominantly in the frontal and parietal bones. CT scan consistently revealed hyperostosis and MRI dural enhancement. All patients underwent craniectomy and complete excision, with four requiring cranioplasty. Histopathological analysis confirmed WHO Grade I Meningioma in all cases. No postoperative complications or recurrences were noted during 3-6 months of follow-up. The study underscores the importance of early diagnosis and complete surgical resection in managing IM and contributes to the limited existing literature.

## INTRODUCTION

Intraosseous meningiomas (IM) represent a rare subset of primary extradural meningiomas that arise within the bone (usually of the skull), from ectopic arachnoid cap cells or meningothelial cells trapped within cranial bones during embryogenesis or after trauma, rather than from the meninges, and account for less than two percent of all such tumors.^1^ Despite being classified as benign lesions, intraosseous meningiomas can exhibit aggressive behavior, particularly in their osteolytic forms, which are associated with a higher incidence of malignant features compared to their intradural counterparts.^2^ The clinical presentation of IM is often insidious, with patients frequently presenting with painless expansile masses, which can lead to delays in diagnosis and treatment.^3^ This highlights the importance of understanding the clinical and radiological characteristics of these tumors to facilitate early detection and intervention.

Radiological imaging plays a crucial role in the diagnosis of intraosseous meningiomas. Computed tomography (CT) and magnetic resonance imaging (MRI) are essential tools for evaluating the extent of the tumor and its relationship with surrounding structures.^4^ IM can present with either an osteoblastic or osteolytic appearance on imaging, with the osteoblastic type being more common and typically associated with hyperostosis of the affected bone.^2^,^5^ Conversely, osteolytic lesions are often more aggressive and may mimic other conditions such as fibrous dysplasia or metastatic disease, complicating the diagnostic process.^1^,^6^ The differentiation between these entities is critical, as the management strategies and prognoses differ significantly.

Surgical management remains the primary treatment modality for intraosseous meningiomas, with the goal of complete resection to minimize the risk of recurrence.^7^,^8^ However, the surgical approach can be challenging due to the tumor’s location and its potential involvement with surrounding neurovascular structures. Recent advancements in surgical techniques, including the use of intraoperative navigation and imaging, have improved the safety and efficacy of these procedures.^9^ Nonetheless, the risk of complications, such as neurological deficits and infection, remains a concern, necessitating careful patient selection and preoperative planning.^10^

In the context of a tertiary care hospital in Pakistan, exploring the clinical features, imaging characteristics, and surgical outcomes of intraosseous meningiomas can provide valuable insights into this rare entity. Local studies are essential for understanding the epidemiology and behavior of these tumors within specific populations, as variations in genetic, environmental, and healthcare factors may influence their presentation and outcome.^11^ Furthermore, by analyzing case series and correlating clinical findings with histopathological results, healthcare providers can enhance their diagnostic accuracy and treatment strategies, ultimately leading to improved patient care. The rationale of this study is to evaluate the surgical outcomes of these patients compared with the previous literature and postoperative management in tertiary care centers in Pakistan.

## CASE PRESENTATION

This study was a retrospective case series conducted at the Department of Neurosurgery Unit-III, Punjab Institute of Neurosciences (PINS) from January 2022 to December 2024. Due to the rarity of intraosseous meningioma, a non-consecutive purposive sampling method was employed. All five available cases were aged 18 years or older, diagnosed with intraosseous meningioma based on radiological and histopathological confirmation, and underwent surgical intervention, with complete clinical, radiological, and histopathological data included in this study. Data was collected from patients’ medical records and Picture Archiving and Communication System (PACS). Variables included demographics, clinical and radiological findings, and surgical outcomes. A structured questionnaire was designed on Google Forms to collect data.

### Ethical Approval:

This study was reviewed and approved by the Institutional Review Board of our Institution with reference no. 2039/IRB/PINS/Approval/2025, dated January 23, 2025.

This retrospective case series included five patients diagnosed with intraosseous meningioma (IM) at a tertiary care hospital in Pakistan. The cohort demonstrated a marked female predominance (four females, one male), with a mean age of 43±15.77 years (range: 19–56 years). All patients presented with a gradually progressive, painless swelling of the craniofacial region, except one (21-year-old female) who reported associated localized pain. Symptom duration ranged from 6 months to 5 years. Lesions were distributed across the frontal (2), parietal (2), and temporal (1) regions (Table-I).

**Table-I:** Demographics with radiology, differential diagnosis, and surgery for intraosseous meningioma.

Age (Years)	Gender	Symptoms	Radiology	Differentials	Surgery
56	Female	Gradually progressive swelling over the right frontal region for five years.	Hyperostosis and bony erosion on CT, dural enhancement on MRI.	Intraosseous meningioma, fibrous dysplasia, osteoma, metastatic lesions.	Craniectomy and excision
21	Female	Gradually progressive swelling over the right temporal region with pain for six months.	Hyperostosis on CT, dural enhancement on MRI.	Intraosseous meningioma, fibrous dysplasia, osteoma, metastatic lesions.	Craniectomy and excision followed by cranioplasty with PMMA bone cement.
19	Male	Gradually progressive swelling over left frontal region for two years.	Hyperostosis on CT, dural enhancement on MRI.	Intraosseous meningioma, fibrous dysplasia, osteoma, metastatic lesions.	Craniectomy and excision followed by cranioplasty with PMMA bone cement.
44	Female	Progressive swelling over the right parietal region for one year.	Hyperostosis and bony erosion on CT, dural enhancement on MRI.	Intraosseous meningioma, fibrous dysplasia, osteoma, metastatic lesions.	Craniectomy and excision, and cranioplasty with PMMA bone cement.
30	Female	Gradually progressive swelling over the right parietal region for two years.	Hyperostosis on CT, dural enhancement on MRI.	Intraosseous meningioma, fibrous dysplasia, osteoma, metastatic lesions.	Craniectomy and excision followed by cranioplasty with PMMA bone cement.

**Fig.1:**
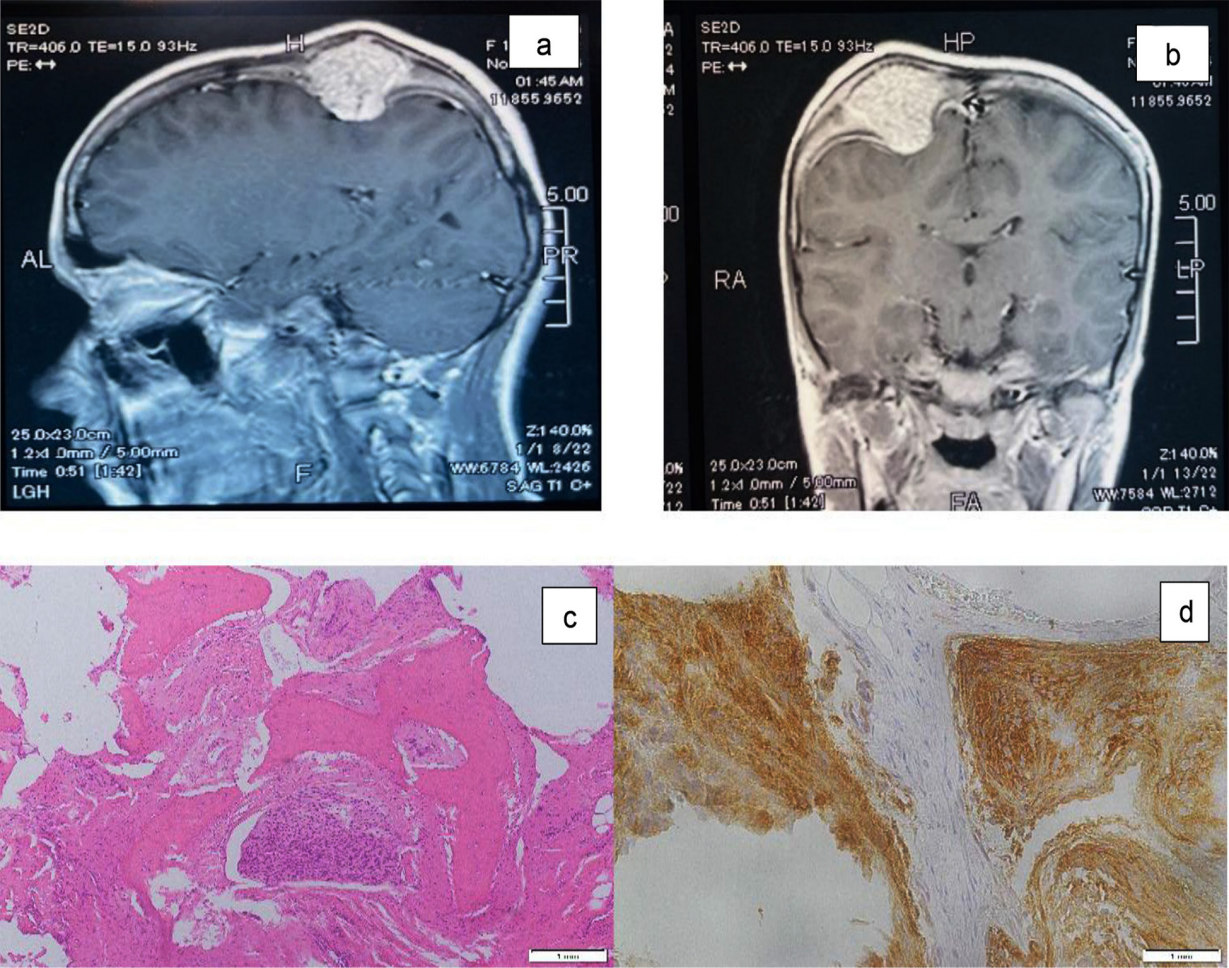
a) shows T1 with contrast showing a homogenously contrast-enhancing lesion at the vertex, and b) shows T1 with contrast showing a contrast-enhancing lesion at the right parietal region. c) shows Sections revealed mature bone trabeculae with interspersed whorls of meningothelial cells showing round nuclei and moderate eosinophilic cytoplasm, and d) Immunohistochemical staining showed positive staining of somatostatin receptor (SSTR2), supporting the diagnosis of intraosseous meningioma.

Preoperative imaging with computed tomography (CT) and magnetic resonance imaging (MRI) revealed consistent features. CT scans demonstrated hyperostosis in all cases, with bone erosions in two patients. MRI complemented these findings by showing adjacent dural enhancement, confirming the extra-axial nature of the lesions (Fig.1a and Fig.1b). Notably, none of the cases exhibited intracranial parenchymal invasion, supporting the diagnosis of primary intraosseous meningioma. The clinical and radiological differential diagnoses included fibrous dysplasia, osteoma, and metastatic lesions. These were systematically ruled out through imaging characteristics and confirmed postoperatively via histopathology (Fig.1c and Fig.1d).

**Fig.2:**
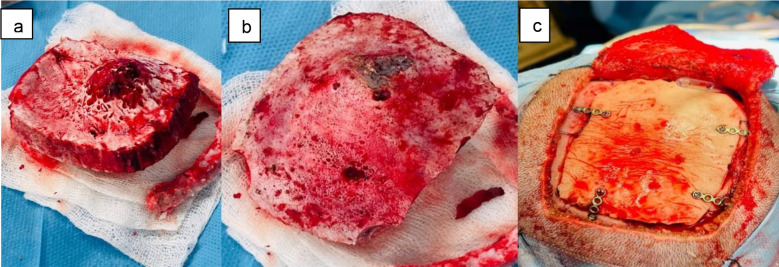
(a and b) show internal and external portions of the excised skull tumor of the right parietal region. (c) shows a bony defect filled with Bone cement (PMMA) cranioplasty and stabilized with plates and screws.

All patients underwent craniectomy with complete excision of the lesion (Fig.2a and Fig.2b). Four out of five patients had additional reconstruction using polymethylmethacrylate (PMMA) bone cement (Fig.2c).

No immediate postoperative complications—such as infection, neurological deficits, or hematoma—were observed. Histopathological examination confirmed WHO Grade I meningioma in all cases, without atypical or malignant features. Short-term follow-up (3–6 months postoperatively) revealed no clinical or radiological evidence of recurrence. Long-term follow-up data were unavailable due to the study’s retrospective design and limited patient compliance.

## DISCUSSION

Intraosseous meningiomas (IM) are rare tumors that arise from the meninges and primarily affect craniofacial bones. Our case series, conducted at a tertiary care hospital in Pakistan, yields substantial data on the clinical presentation, radiological characteristics, surgical outcomes, and differential diagnoses associated with IM. The study included five patients with a mean age of 34 years, demonstrating a female predominance, which is consistent with the demographic trends reported in the literature.^12^ The key findings indicate that patients typically present with gradually progressive swelling, with imaging studies revealing hyperostosis or lytic changes and dural enhancement. The clinical presentations observed in our series are consistent with previous literature, which underscores the insidious nature of IM. Patients often report painless expansile masses, leading to delays in diagnosis and treatment.^13^,^14^ The imaging characteristics noted in our study, including hyperostosis and lytic changes on CT, align with findings from other studies that emphasize the importance of radiological evaluation in differentiating IM from other conditions such as fibrous dysplasia, osteoma, and metastatic lesions.^15^ The differential diagnoses we identified echo those reported in the literature, underscoring the complexity of accurately diagnosing IM based solely on imaging. Surgical resection is indicated for symptomatic intraosseous meningiomas, particularly when tumors are progressive or cause functional deficits or cosmetic deformity.^16^ Surgical indications for intraosseous meningiomas in our series included: progressive enlargement causing cosmetic disfigurement, symptomatic mass effect with localized pain, radiological features suggesting potential for aggressive behavior (bone erosion), and diagnostic uncertainty requiring histological confirmation. Surgical management remains the cornerstone of treatment for IM, with the goal of complete resection to minimize recurrence risk and rule out malignant transformation. In our series, all five patients underwent Simpson Grade-I resection involving complete gross total removal of the tumor, dural attachments, and abnormal bone, with four patients also receiving cranioplasty using PMMA bone cement. It was feasible due to bone-confined tumors without dural extension, en bloc craniectomy removal, absence of brain invasion, and wide surgical margins achieved in every case. This approach is consistent with the recommendations from previous studies, which advocate for aggressive surgical intervention in cases of IM.^6^,^9^ Our outcomes compare favorably with an international study. Literature reports recurrence rates of 12.6-22% for incompletely excised intraosseous meningiomas.^17^ Our achievement of Simpson Grade I resection in all cases suggests excellent short-term prognosis, although long-term follow-up with MRI is necessary for definitive assessment of recurrence rates. International studies suggest that osteolytic variants may have higher recurrence potential, emphasizing the importance of complete bone removal as achieved in our series.^5^

The significance of our findings lies in their implications for clinical practice and medical research. Understanding the clinical and radiological features of IM can facilitate early diagnosis and intervention, which is critical given the potential for aggressive behavior in certain cases.^3^ Furthermore, our study contributes to the limited literature on IM in the Pakistani population, providing insights into its epidemiology and behavior in a specific demographic context. This localized understanding is essential for tailoring management strategies and improving patient outcomes.^5^

## CONCLUSION

Intraosseous Meningiomas, a benign pathology of the skull, have a female predominance, typically causing scalp swelling without neurological symptoms. However, they can become invasive without/delay in treatment. CT shows hyperostosis while MRI reveals dural enhancement, both crucial for surgical planning. Complete surgical excision via craniectomy, followed by cranioplasty, yields excellent outcomes with reduced recurrence rates and good cosmetic results for IM’s histopathology of WHO Grade-1 Meningioma, underscoring the importance of immediate skull reconstruction post craniectomy.

### Clinical recommendations:

Future research should focus on larger, multicenter studies to validate our results and explore the molecular and genetic underpinnings of IM, innovative treatment modalities that could enhance patient care. Additionally, investigating the long-term outcomes of patients’ post-surgery could provide valuable insights into recurrence rates and the effectiveness of different surgical approaches.

### Author`s Contribution:

**NA:** Concept of the study, critical review of the manuscript, and supervision.

**SM:** Critical review and revision of the manuscript.

**SSHR AUR TS AM:** Data acquisition, drafting, and critical review the manuscript.

All authors have approved the final version to be published and agreement to be accountable for all aspects of the work in ensuring that questions related to the accuracy or integrity of any part of the work are appropriately investigated and resolved.
